# Westminster Hospital

**Published:** 1830-01-01

**Authors:** 


### CLINICAL REVIEW.

### XIV.

### WESTMINSTER HOSPITAL.

I. FRACTURE OF THE STERNUM, CLAVICLE, EIGHT RIBS, AND COMPOUND FRACTURE OF THE ULNA.—DIFFUSED INFLAMMATION OF THE SCALP—TREATED BY INCISION.

John Burkett, æt. 45, admitted October 11th, 1828, under Mr. Guthrie, was brought into the hospital in consequence of a mass of bricks having fallen upon him whilst assisting in taking down a house in St. Martin's Lane. On examination there appeared a lacerated wound of the scalp about four inches in length extending along the right parietal and frontal bones. The sternum fractured about two inches from its superior extremity, the left clavicle fractured in two places, five ribs on the right and three on the left were also broken—those on the right side were in addition separated from their cartilages near the sternum. The ulna of the right arm broken about its middle and a piece splintered off about half an inch from its proximal extremity. The olecranon laid bare, and also the external condyle of the humerus.

Pulse 60, feeble—extremities cold—℥iss. brandy statim. The wound on the head approximated with stickingplaster and bandage—the figure of 8 bandage to his shoulders, and a flannel roller to his body. The edges of the wound on the elbow brought together with sutures, &c. and the arm put in splints. There is a considerable extravasation of blood extending from above the fractured clavicle to as far as the fourth rib, and also a slight wound from which a little blood oozes out.

Two hours after admission the pulse continuing feeble, some wine was given to him and continued every hour—complains but little of pain.

8. p. m. Complains of pain in his head — pulse stronger — extremities warm, omit the wine. ℞. Pil. Aper. ℈ss. statim sumend.—Mist. Purgantis, ℥ij. mane sumend. et repetantur 4tâ quaque horâ donec alvus respondet.

12th. *Mane.* Has had some sleep in the night — considerable pain in the head, respiration hurried and performed with pain. Pulse 96, full. Detrahetur e brachio sanguis ad ℥xiv—pulse 100, much reduced in strength.

*Meridie.* Bowels have been freely evacuated—better.

*Vespere.* Suffers but little pain—pulse 90, rather weak.

13th. Mr. Guthrie saw him this morning—on removing the splints from the arm it appeared swollen.—Applicentur Hirudines xij.—appears to be going on well.

*Vespere.* Bowels have been once acted upon during the day. — Repetantur Pilulæ, &c.

14th. Slept a little during the night — pulse 134, soft — skin moist. The dressings removed from the head—the wound nearly united by the first intention. There is, however, a little puffiness—the wound was therefore laid open in part of its extent.

*Vespere.* He answers questions sharply—pulse small, wiry—Rep. Med. Aper.

15th. He has passed rather a restless night and at times spoken incoherently.—This morning he is better—bowels freely open.

*Vespere.* He is become very incoherent, and is indeed delirious—restless and with difficulty kept in bed. On examining the scalp which had been shaved, it was found in a state of general puffiness causing the head to look considerably larger than usual, but without a shade of redness—it retained the mark of the finger—pulse 98, wiry.—Mr. Guthrie on examining the head, said the case was one of diffused inflammation of the cellular texture both above and below the occipito-frontalis tendon, constituting a very rare example of disease, being in fact the same as that which in other parts was frequently called erysipelas phlegmonodes. The erysipelatous flush being alone wanting and therefore shewing that it was not an essential sign of the complaint. The only practice which could save the patient's life was, he said, by incision so as to relieve the internal and external parts at the same time, by which the disturbance and the general irritation would be removed, and he recalled to the recollection of one of the gentlemen present the case of Thomas Key, treated in the hospital, in 1823, and which he had published with his observations on erysipelas, in his work on Gunshot Wounds. That case he said led the way to the treatment of diffused inflammation by long incisions, and was published before any others were treated in that manner.

The present was a well-marked one of the same nature, and the only hope of safety was by a similar mode of treatment. He made two incisions in the scalp on each side of the sagittal suture —one six inches long, the other four—united them by a cross cut and added another from the ear upwards. The scalp was upwards of an inch in thickness and filled with a serous and partly purulent fluid which was squeezed out in all directions. Several arteries bled freely and were allowed to do so until he was presumed to have lost about thirty ounces of blood, when the hæmorrhage was stopped by pressure. The man became more calm. The head was directed to be fomented.

16th. The patient was restless during the night but is more composed, and sensible—and greatly relieved.—Pulse only 84 and small.—There is a manifest improvement in the scalp. Mr. Guthrie said the imminent danger was overcome as regarded the scalp, for he considered the man would not have survived this day if any other mode of treatment had been adopted. Bowels open—to continue fomentations.

17th. Has had some sleep in the night and this morning is much more tranquil—there is a discharge of healthy pus from the head.

19th. Has slept well during the night — the bowels have been very freely acted upon—motions dark coloured and fetid.

℞. Pulv. Rhei. ℈j.

Aquæ Menth. Pip. ℥iss. statim sumendus.

He was ordered yesterday—
Quinine Sulph. gr. i.
Acid. Sulph. d. gtt. x.
Infus. Rosæ, ℥iss. ter. in die sumend.

20th. Rested well—free discharge of pus—dressed and a bandage applied—pulse 104.

*Meridie.* Mr. Guthrie made another incision at the back of the head and also at the side.

22nd. Free discharge of healthy pus from the head—pulse 100—rather feeble—to take nourishing diet.

From this time he continued improving—a large abscess was formed where the clavicle was fractured—it was laid open and a great quantity of matter discharged.—The elbow healed up.—The incisions on the head after a time were united.—The ribs too became firm though it may yet be counted how many were fractured. About four months ago a shell of bone of about an inch in length was thrown off the clavicle, and three months back when he was discharged, he had tolerably good use of his arms and was in perfectly sound health.

### II. Lacerated Wound in the Bend of the Arm.

Mary Norman, æt. 15, admitted August 26th, under Mr. Lynn. The patient was brought in having fallen down with a jug in her hand, one of the fractured portions of which had pierced her left arm and made a deep lacerated wound in the bend immediately in the triangular space.

On removing a handkerchief which had been tied tightly across, there were two or three gushes of blood, which ceased as soon as pressure was made over the artery above. A tourniquet was applied, the wound a little enlarged downwards and cleaned—it then appeared that the basilic and median basilic veins had been divided, and from them had gushed the greater part of the blood. The median nerve was wounded, but on loosening the tourniquet no hæmorrhage ensued and it was ascertained that the humeral, radial, and ulnar arteries were uninjured—they could all be felt to pulsate by placing a finger in the wound.

The edges of the wound were brought gently together — a compress placed over, and bandage secured it. She was said to have lost a considerable quantity of blood before she arrived. She was rather hysterical during the dressing—pulse very faint—she had two ounces of port wine and half a drachm of the Spt. Æth. Sulph.

She was taken to bed immediately afterwards, and on seeing her half an hour later she complained of great pain in her arm—she was ordered a cold lotion to the arm.

Aug. 27th. She feels but little pain in the arm—pulse 90—ordered a powder of rhubarb and calomel.

28th. The dressings were removed this morning, the wound remained closed but not healed. She complains of great pain up to her arm-pit—the whole arm is swelled and hot. A strip of plaster is put across and a large poultice ordered to be applied—pulse rather quick—tongue clean.

29th. The arm is still swelled and hot but less painful—pulse 124, small and wiry—tongue pale—bowels open.

Sept. 10th. The inflammation and fever has gradually subsided—her arm is its natural size, freer from pain and heat, but slightly hardened along the course of the vessels. She cannot supinate or straighten her arm beyond the semiflexed position, in which it has been kept since the accident—the wound is nearly healed.

### CLINICAL REVIEW.

### XXXVII.

### WESTMINSTER HOSPITAL.

#### I. FATAL INJURY OF THE KNEE-JOINT.

George Budd, æt. 52, admitted August 24th, 1829, under Sir Anthony Carlisle; states that ten days ago he was shaping a pole with an axe, and missing one of his strokes, he struck his right knee on the inside—it did not bleed much, and he bound it up and was carried home. The next morning he had medical advice, and his knee was strapped and bandaged; he was not seen again until two days after, in the mean time he had suffered great pain, and his knee and leg became red and swelled. Poultices were then applied, and continued until he was admitted yesterday. Thirty leeches are ordered to be applied; fomentations, and the following mixture:

℞. Infus. sennæ, . . . ℥viij.

Magnes. sulph. . . ℥j.

Potassæ supertrart. ʒiij.

Sodæ carb. . . . . . ʒj.

Ft. mist. et capt. cochl. ij. 4tis horis.

Aug. 26th. On examining the knee this morning it is found to be considerably swelled, red and hot, and he complains of great pain in it. His tongue is pale and slightly furred—bowels not opened since yesterday morning. On pressure a considerable quantity of serous fluid with flakes of white matter escaped from the wound. Skin moist and cool—pulse 96 in the minute, small and thready.

℞. Hydr. submur. gr. v.
Pulv. jalapæ, . ʒss.

Ft. pulv. st. sumend.—Fomentations of poppy-heads, &c. to be continued.

Aug. 27th. The fomentations have been constantly applied—he does not complain of so much pain, and his knee seems altogether in a quieter state. As he has been lying so as to allow the discharge to escape by the wound, there was not much observed flowing out this morning. Pulse full and 90 in the minute—bowels opened once yesterday—tongue pale. Rep. pulv. purg.

Aug. 28th. The heat and pain in the knee are diminished; there appears also to be less discharge. Fomentations to be continued. Pulse quick, not very strong.

Aug. 29th. Pulse 96, hard, and rather wiry—tongue clean—the knee is apparently much the same. He says he suffers considerably less pain than he did two or three days back. Bowels open.

Sept. 10th. The patient has been going on very slowly but progressively improving, the knee has become quieter—the discharge less, and the wound healed up to a mere point, from which a very small quantity of discharge oozes. His bowels are open, he complains that he is weak, suffers but little pain, and that is chiefly confined to the ham.

Sept. 16th. His wife insists on taking him out—he is much the same.

Sept. 30th. Information was brought that he died two days ago.

II. Sympathetic Bubo, attended with a peculiar Eruption.

Samuel Stokes, æt. 15, admitted under Mr. Guthrie, October 7th, 1829. A fortnight previous to his admission he was lifting a heavy cask of beer and strained himself greatly—he rested the edge on the right groin. The next day he felt the effects of the straining in his back, and whenever he attempted to walk had great pain in his right groin. He went to bed, and three days afterwards he observed a swelling in his right groin. He was bled, and had some medicine; and the principal effects of his exertion in a short period went off, but the swelling still remained increased in size and hardness.

This patient also suffers from an affection of the heart, which has existed since childhood—its palpitations are so strong that he says he feels it "ticking" in his neck—he has great oppression at the chest, with difficulty of breathing. The action of the heart may be felt very distinctly all over the chest and along the spine. He has often great difficulty in getting up stairs.

℞. Hyd. submur. . gr. iij.
Pulv, jalapæ, c. ʒss.
Ft. pulv. st. sumendus.—Poultice to the tumour.

10th. Yesterday evening he observed an eruption on his skin, and this morning he is covered with a copper-coloured eruption. Tongue pale, skin hot and dry. Bowels not very open.

℞. Liq. ammon. acet. d. ℥iij

Liq. ant. tart. . . . . ʒiij.

Mist. camph. . . . . ℥v.

M. et cap. cochl. ij. 4tis horis.

11th. He is better to-day—less feverish—the eruption is rather stronger in colour—the swelling in his groin is larger and softer.

12th. He continues better—eruption the same.

14th. The fever has left him—the eruption is fading.

17th. The spots have gradually assumed a fainter tint—they are still of a well-defined copper colour.

19th. Pulse 100, moderately strong, action of the heart excessive. The eruption has disappeared, leaving copper-coloured stains. Tongue clean—bowels open. He is free from fever, but complains of tightness across his chest. The tumour in the groin has been rubbed with the potassa fusa. Acid. hydrocyanici, ♏j. ter in die sumend.

25th. The abscess in the groin now presents a clean ulcerated surface—his chest is better, and his general health improved.

27th. He complains of pain in his chest. Hirudines xviij. applicand.

30th. Pain still continues. Rep. Hirudines.

31st. He is better this morning—pulse 100, soft.—skin hot and rather dry—tongue clean—bowels open. Acid. hydrocyan. ♏ij. 4tis horis, sumend.

Nov. 8th. The ulcer in the groin is healing—the eruption and stains have entirely disappeared.

III. Diseased Testicle.

William Cox, æt. 53, admitted June 20th, 1829, under Mr. Guthrie. States that about seven years ago, he thinks, in consequence of a strain, he had the right testicle enlarged. It appeared much inflamed, greatly increased in size, and so painful that he could not stand upright. He had medical assistance—some veins were opened on the scrotum, and he thinks he lost about a pint of blood, and he felt relieved by it. After being confined to bed for six weeks with cold lotion to the part, &c. he was able to get up and go to his work. He has worn a suspensatory bandage ever since. The testicle returned to its natural size, and he felt no inconvenience from it until February last, when it again became swelled and painful without his being able to assign any probable cause. This attack confined him to bed for six weeks, having cold lotions, medicine, &c.—he then proceeded to work for three weeks, when he was again obliged to return to his work—he had cold lotion applied for about a month, and after this a liniment. He remained for about eight weeks unable to do any work, and was then admitted into this hospital.

On examining him fluid was felt, which Mr. Guthrie let out by an incision with a lancet. The testicle was then more distinctly perceived to be hard, elongated, and knotty—but without much pain.

This patient is not of very strong constitution, but generally enjoying good health, except during the last five years that he has been much troubled with rheumatism in all his joints. He never had gonorrhœa but once, about 15 years ago, which was slight and speedily cured—he has never had syphilis and lived temperately, not much addicted to excess of any kind.

He was ordered a few days after his admission the following powder:—

℞. Hyd. submur. gr. iij
Pulv. conii, . ℈j.

M. et divide in partes vj. et cap. j. ter in die.—Conii folia pro cataplasm.

July 3d. He had the poultice of fresh hemlock leaves mixed with sufficient of linseed meal to make it of good poultice consistence applied yesterday—to continue.

July 15th. His mouth has become sore—the testicle is diminished in size, much smoother. Ten grains of pulv. conii to be added to the powders. Full diet.

July 24th. Tincture of iodine is ordered to be applied to the scrotum until it produces blistering. Powders discontinued—he has evidently derived much benefit from the poultices.

October 15th. He has had the testicle blistered in this manner five times, allowing an interval of some days to elapse between each; he has also had a large sound passed twice a week to excite a little irritation. The testicle is now very much diminished in size, perfectly smooth, and quite free from pain.

Oct. 23d. Blister repeated.

29th. There is evidently fluid within the tunica vaginalis, which Mr. Guthrie proposes letting out. The testicle does not appear much above the natural size.

Nov. 8th. Mr. Guthrie made a puncture with the lancet, and about an ounce and a half of fluid escaped. The testicle was then examined, and ascertained to be smooth, and not above the natural size. He suffers no pain.

---

### XXXVIII.

### ROYAL WESTMINSTER OPHTHALMIC HOSPITAL.

### WESTMINSTER HOSPITAL.

I. Medullary Sarcoma in the Ham—Affection of the Lungs and Liver.

Amelia Beckett, æt. 57, admitted, Nov. 3d, 1829, under Mr. Guthrie. States that two years ago, last month, she perceived a lump in the lower part of her left leg—hard, immoveable and free from pain, and is certain there was never any pulsation in it. The tumour gradually increased, and, two months afterwards, was about the size of a large orange, when a lancet was pushed into it, a little below the origin of the gastrocnemius, and a few drops of blood was the only result. She has at intervals suffered great pain from it—sometimes shooting through the swelling, extending down to the heel, accompanied with a tingling feeling in the sole of the foot and round the ends of the toes. About four months ago she had plaisters applied—then linseed meal poultices, and, lastly, fomentations. She describes herself as having been always healthy, and, previous to this disease, a strong stout-looking woman. She is now very much emaciated.

On examining the left leg, we find the whole limb œdematous—pitting on pressure—a great and tense enlargement beginning in the popliteal space, just above the origins of the gastrocnemius, extending forwards and downwards on each side—rather flattened on the outside, more projecting and rounded on the inside; its origin is perfectly immoveable, and apparently unconnected with the tendons forming the hamstrings—it occupies the whole extent of the gastrocnemius. The veins ramifying over it are all distended, and in a varicose state up a considerable portion of the thigh—the tumour is hot, and at one or two different points has an elastic feel. The diseased leg is more than twice the size of the other—moderate pressure does not cause her any pain. She suffers considerably from a cough—her pulse is feeble and quick, and she is evidently much exhausted by the continuance of the disease. She is ordered fish and mutton-chop, and a pint of porter daily.

It was evident that amputation offered the only chance of success in such a case as this, although it was more than probable, Mr. Guthrie stated, that the lungs or other internal part were affected, as appeared to be shewn by the cough and irritation under which she was labouring, and, if so, it would then be rendered unavailing. A consultation having been held, these circumstances were explained to the woman, who expressed her willingness that the operation should be done.

Mr. Guthrie, in proceeding to the operation, stated that there were some peculiarities in his method of performing amputation of the thigh which he should explain—1st. That he never applied the tourniquet, when assistants could be obtained, on whom he could depend, for compressing the artery against the pubis—he only made use of it when proper assistants were not at hand, because great opportunities of observation had convinced him that there was much less blood lost when the tourniquet was not employed; the strap very often interfered with the operation and prevented the necessary retraction of parts, as well as gave rise to other inconveniences. The next point was, that the first incision should go through the fascia lata, the points of attachment to which running between the muscles on the outside and posterior part of the thigh, were also to be divided, so that skin, integument, and fascia might be drawn up by the assistant, without any of that tedious dissection of the skin backwards, which was as unnecessary as it was painful. Lastly, that in a very thin person, such as this, the muscles, after being divided, should be so far retracted, that three inches and a half, or even four inches of bone should be exposed before the saw should be applied, and which should always be held in a perpendicular direction.

The operation was performed on these principles in 80 *seconds*, two arteries only being tied. No blood was lost.

The patient bore the operation remarkably well, and appeared to be going on favourably for the first few days, when an irritative fever set in, with an increase of cough and expectoration, and she gradually sunk until the 14th day after the operation, when she died.

On the day succeeding the operation, the arteries and veins of the amputated limb were injected with wax, an incision was made from the popliteal space in the center, between the two portions of the gastrocnemius, into the tendo Achillis.

Immediately on getting through the fibres of the muscle a considerable quantity of yellow pultaceous fluid escaped—probably about half a pint. On enlarging the opening and clearing it, it was evident that the knife had divided a tumour of considerable size, the left side of which was about double the size of the right—the upper portion was excavated and the cavity filled up by the curdy pultaceous matter, which was more fluid where the elastic spring had been felt during life.

The walls of the tumour were formed by a partly cartilaginous, partly lardaceous, substance, thicker in some parts than others. The popliteal nerve was increased in size, and passed under the tumour, between it and the bones, having been pressed upon by it, accounting for the pain experienced in the toes. Just as the artery passes down from the popliteal space, two arteriæ surales, nearly three times the natural size, were given off, passing along the outer surface, between the fibres of the gastrocnemius, and fulfilling the duty of posterior tibial and fibular arteries, which were obliterated. The internal edges of the tibia and fibula were both roughened, and in some degree softened, evidently the result of the pressure.

The post-mortem examination took place four days after death. On opening the thorax, the lungs were found to have several large tubercles in the different lobes, which, when cut into, shewed the same white medullary-sarcomatous appearance evidently connected with the disease in the leg. The liver was also similarly affected. On tracing the veins up into the pelvis, they were found inflamed as far as the common iliac. A model of the leg, shewing the dissection of the tumour, has been made, and may be seen at the hospital.

II. Ulceration of the Urethra—Extravasation of Urine—Scarifications—Death.

George Huntley, æt. 1 year and 9 months, admitted, December, 2d, under Mr. Lynn. On Sunday last, Nov. 29th, the child cried on voiding its urine, and on Monday, at two o'clock, flinched and cried again excessively. The quantity voided was less than a cupful and passed in a full stream. On Monday evening he appeared to suffer great pain in making water, which was only expelled by drops. He was seen by a medical man and some medicine ordered—during the night the scrotum swelled—was red and tense, and the patient was restless and appeared to suffer much pain. On the Tuesday he was fomented, and the whole of the scrotum and penis scarified with the lancet: on Tuesday night and Wednesday morning he appeared in less pain.

Wednesday, Dec. 2. He has passed no water since Monday morning—the scrotum is distended, semi-transparent, of a pale lake colour, and evidently painful on pressure—bowels open. Mr. Lynn has made several punctures in the scrotum with the lancet, from which some fluid has oozed out. Fomentations ordered to be continued.

3d. Mr. B. Lynn endeavoured, by means of bougies, to find the orifice of the urethra, but without success—the prepuce is very much elongated, for it allowed the bougie to pass an inch and a half before it was stopped. About 6 ounces of urine were ejected during the attempt, and a small calculus, about half an inch in length and an eighth in thickness, pressed out of the urethra. Fomentations were ordered to be continued, and ℥ss. of the ol. ricini to be taken immediately. He has passed a tolerable night and the flannels have a urinous odour. Bowels have been freely opened. The tongue is clean—pulse very feeble. Scarifications repeated.

4th. Ulceration has taken place about half an inch from the end of the prepuce. The urine passes both through this opening and the end of the penis. The scrotum is distended, and at the lower part is assuming a deeper tint—the child seems more fretful and passed a restless night.

5th. The scrotum is looking better—the distention has in some degree subsided, and the urine passes freely by the natural orifice.

6th. Passed a very restless night—bowels confined—tongue dry—skin hot, shewing an attack of low fever.

℞ Pulv. ant., gr. ij.
Hyd. submur., gr. ij. ft. pil. st. sumend.

7th. He had a cretaceous mixture with xv. minims of tinct. opii, last night, as he was suffering from an attack of diarrhœa—he was in convulsions at one part of the night, and early this morning died.

The friends were averse to an examination—but the urethra and scrotum were opened—the urethra appeared to have been ruptured a little above the membranous portion. The cellular texture in the scrotum was in a sloughing state—the bladder was slightly diseased, but no calculus or gut found in any part of the passage or bladder.

### III. Compound Fracture of Skull, with Wound of the Brain.

Thomas Sullivan, æt. 15, admitted, Dec. 4th, under Mr. Guthrie. This patient was brought in this morning, having fallen 40 feet in height. He was in a state of insensibility, and, on examining his head, it was found much swelled at the posterior lateral part, and a wound, about two inches in length, extending across nearly in the direction of the lambdoidal suture. On passing the finger into the wound, an extensive fracture was felt in that direction, with considerable depression. His breathing was stertorous—pupils dilated and insensible to light—considerable hæmorrhage from the ear—pulse imperceptible—a tea-spoonful of brandy was administered at short intervals and he was somewhat revived, and his pulse was 140, very small, trembling, and intermittent—skin cold.

When Mr. Guthrie arrived he had vomited twice, and about a tea-spoonful of brain was forced out; his extremities were rigid. He immediately enlarged the wound by incisions in two or three directions—it was then seen that the fracture extended for several inches both forward and downward, and a large portion of the parietal bone was depressed and overlapped by the adjoining bone. This depression was relieved by sawing off the angles with Hey's saw, but it was now found that the fracture extended in every direction, having separated the mastoid process from the temporal bone, leaving space for the finger to pass between. All further attempts were, therefore, given up. The patient appeared relieved when the bone was raised, and lived until 8 o'clock in the evening. During the operation of raising the portion of bone, Mr. G. pointed out the use of an instrument which enabled him to draw up the depressed portion, so as to counteract the inclination it had to return, without making pressure on any other part. During the operation nearly a tea-spoonful of brain escaped out of the ear.

On examining the head after death, one side of the brain was completely beaten in, and a considerable portion of the substance lost. The brain was much injected, and there was no coagulum in the base. The fracture had extended into the base of the skull separating the petrous portion of the temporal bone, and stopping at the lesser ala of the sphenoid bone.

### IV. Psoriasis Diffusa.

Mary Macartey, æt. 14, admitted October 28th, 1829, under Mr. Guthrie. States that six weeks ago she was running and fell against a wall grazing the skin about the situation of the right trochanter major. This scabbed and peeled off after a time leaving large red and scaly patches. She had not observed any eruption before this, but says that during the succeeding fortnight she observed it spreading across the loins unattended with pain or any further inconvenience than a slight itching at intervals. She had no medicine or applications for it until she first attended at the hospital five days ago.

She has not yet menstruated; she had the small-pox when about three years old and the scarlet fever five years back. She has been out of health ever since—suffering from continued pain in the head attended with drowsiness. She has never before had a similar eruption—bowels have been generally in a confined state.

She is pale, thin, and altogether of sickly appearance. The whole of her loins—each side of the hips—her knees, left shoulder, and fingers of both hands are covered with large continuous blotches — in some parts the skin is smoothed and red, except where it is divided in furrows, some of which are covered with whitish scales slightly elevated.

Pulse natural— tongue clean—bowels confined. Ordered warm bath to-night and the following medecine.

Pil. Hyd. c. Col. gr. x. ft. pil. ij. st. sumend. Haust. Purg. ℥ij. mane sumend.

31st. Rep. baln. tepid. et medicinæ.

Nov. 2. Bowels not freely opened.

℞ Hyd. Submur. gr. v.

Pulv. Jalapæ, ℈j. ft. pulv. st. sumend.

3d. Her bowels have been freely acted upon—she is ordered to take Liq. Arsen. ♏iv. bis in die.

6th. The eruption is spreading across her loins and shoulders—she continues the warm bath every other night.

9th. She does not improve—the skin is very tender and she complains of great pain in the head.—Bowels open —tongue clean—ordered to take the medicine three times a day, with ♏xv. of the Liquor Potassæ to each dose.

10th. Her head is much better—the eruption still seems spreading.

16th. Her general health is much improved—the eruption is much the same.

18th. The dose of the liquor potassæ is increased to xxxv. minims and vij. of the liquor arsenicalis three times a day.

23d. There is a decided improvement both in general health and the state of the skin, which is less tender — the scales also are peeling off.

28th. Continues improving.

Dec. 1st. All the scales have been thrown off—her hands, hips and shoulders have merely a red appearance left where the scales were—she is made an out-patient and ordered to continue her medicine.

### V. Diseased Penis.

John Mills, admitted Nov. 7th, 1829, under Sir A. Carlisle.

This patient states that it is fifteen years since he first perceived any disease in the penis. It was at a time that he was leading a very irregular life, and he observed some little inflammation about the end of the penis, and after a time a difficulty in drawing back the prepuce. It appears he took very little care of himself, and at last he was unable to draw it back at all, and it has continued in that state ever since.

He also states that he had a discharge from the urethra, two years after the first commencement of the disease, and from not being able to wash the gland it became excoriated, and he thought the prepuce began to feel "horny."

He was in the hospital last year under Sir G. Tuthill for an asthmatic complaint, from which he has suffered for some years, and was then transferred to Sir A. Carlisle.

Mr. Harding's the assistant surgeon, treatment tended to prevent the skin from closing in round the gland to which it shewed a great disposition—to effect this, pledgets of lint dipped in different washes have been introduced between the prepuce and gland, and twice the skin was divided so as to free the gland and lay it bare. It was then seen that the whole surface was covered by a hard unhealthy-looking sore, and that part of the skin adjoining was in a cartilaginous state.

It had become quiet and tolerably free from pain, and the man was dismissed at his own request, thinking he might move about with it, without much inconvenience—he was however re-admitted Nov. 7th. The disease in a very short time had much increased in malignancy — he had much difficulty in voiding his urine, and required a catheter to be passed once or twice each day—the urethra was diseased and much contracted for the first two inches. Amputation seemed to be the only resource, and after a consultation the patient readily consented.

Nov. 10th. Mr. Harding performed the operation to-day, by directing the skin to be drawn back towards the pubis, and then carrying his scalpel perpendicularly down through the substance of the penis, about an inch from the pubis. An even surface was left, and on being allowed to retract, the skin extended well over the edges. Ligatures were put upon three arteries, and very little blood lost.

11th. He seems very easy, only complaining of the smarting when the urine is passed.

15th. He has been going on well—the stump has healthy granulations, and is gradually healing over. He is much troubled with an asthmatic cough—his appetite is not good, but his general health is decidedly improved. He is ordered cough mixture.

18th. There appears to be a disposition in the surrounding parts to contract over the urethra. A bougie is ordered to be passed every day. There is a slight inflammation in one of the testicles.

25th. The inflammation of the testicle has much increased. Hirudines xx. applican.—Haust. cathart. st. sumend.

29th. The testicle is better. The stump is going on well—the asthma distresses him much.

Dec. 7th. The stump has nearly all healed, but the skin seems to have contracted rather too much round the orifice.

